# *Mst1* and *Mst2* Are Essential Regulators of Trophoblast Differentiation and Placenta Morphogenesis

**DOI:** 10.1371/journal.pone.0090701

**Published:** 2014-03-04

**Authors:** Xingrong Du, Yongli Dong, Hao Shi, Jiang Li, Shanshan Kong, Donghua Shi, Ling V. Sun, Tian Xu, Kejing Deng, Wufan Tao

**Affiliations:** 1 State Key Laboratory of Genetic Engineering and National Center for International Research of Development and Disease, Institute of Developmental Biology and Molecular Medicine, School of Life Sciences, Fudan University, Shanghai, China; 2 Howard Hughes Medical Institute, Department of Genetics, Yale University School of Medicine, New Haven, Connecticut, United States of America; University of Houston, United States of America

## Abstract

The placenta is essential for survival and growth of the fetus because it promotes the delivery of nutrients and oxygen from the maternal circulation as well as fetal waste disposal. *Mst1* and *Mst2* (*Mst1/2*), key components of the mammalian *hpo/Mst* signaling pathway, encode two highly conserved Ser/Thr kinases and play important roles in the prevention of tumorigenesis and autoimmunity, control of T cell development and trafficking, and embryonic development. However, their functions in placental development are not fully understood, and the underlying cellular and molecular mechanisms remain elusive. Here, we investigated the functions of *Mst1/2* in mouse placental development using both conventional and conditional (endothelial) *Mst1/2* double knockout mice. We found that the number of trophoblast giant cells dramatically increased while spongiotrophoblast cells almost completely disappeared in *Mst1/2* deficient placentas. We showed that *Mst1/2* deficiency down regulated the expression of *Mash2,* which is required for suppressing the differentiation of trophoblast giant cells. Furthermore, we demonstrated that endothelial-specific deletion of *Mst1/2* led to impaired placental labyrinthine vasculature and embryonic lethality at E11.5, but neither affected vasculature in yolk sac and embryo proper nor endocardium development. Collectively, our findings suggest that *Mst1/2* regulate placental development by control of trophoblast cell differentiation and labyrinthine vasculature at midgestation and *Mst1/2* control labyrinth morphogenesis in trophoblast- and fetal endothelial-dependent manners. Thus, our studies have defined novel roles of *Mst1/2* in mouse placental development.

## Introduction

The placenta is the first organ formed during mammalian embryogenesis to establish the maternal-fetal circulatory system for nutrients and gas exchange as well as fetal waste disposal [1], [2]. Subtle perturbations in its morphogenesis and functions may result in organ malformation, pregnancy complications and early pregnancy loss [3]. The fully developed mouse placenta consists of three distinct layers: the maternally-derived decidua, which directly contacts with the uterus, and two embryonically-derived layers, the junctional zone composed of trophoblast giant cells and spongiotrophoblast cells, and the labyrinth zone, a highly interconnecting structure formed by outer epithelium derived from the trophoblast cell lineage and an underlying vascular network and stroma derived from embryonic mesoderm [3].

Using gene-targeting technology, to date more than 100 mouse models have been reported to exhibit various defects in trophoblast differentiation and/or placenta morphogenesis [1], [3]. Trophoblast giant cells arise from the direct differentiation of mural trophectoderm at the blastocyst stage and later differentiate from the ectoplacental cone cells after implantation. While the differentiation of trophoblast giant cells appears to be a ‘default pathway’, it is regulated by a series of basic helix-loop-helix (bHLH) genes [4]. The bHLH *Mash2* (also known as *Ascl2*) gene suppresses differentiation of trophoblast giant cells (TGC). Deletion of *Mash2* results in premature loss of the ectoplacental cone/spongiotrophoblasts and an increase of TGC differentiation [5], [6]. In contrast to suppressing TGC differentiation, the bHLH *Hand1* and *Stra13* genes can promote TGC formation [7], [8].

The labyrinth is a complex of trophoblast, mesoderm and vascular derivatives. Major labyrinthine morphogenetic events are initiated and regulated by trophoblasts [9]. Multinucleated syncytiotrophoblast cells differentiate from the chorionic trophoblast cells through cell fusion when the chorion begins to fold to form the villi, creating a space into which the fetal blood vessels grow from the allantois [3]. *Gcm1*-positive trophoblast cells define the sites where folding of the chorionic plate and invagination of the allantoic mesoderm occur [10], [11]. Whereas *Gcm1* is essential for chorioallantoic morphogenesis, many other genes including those encoding transcription factors and signal transduction pathway components. regulate the extent of labyrinth growth [2], [3]. A common phenotype among different mutants is that the labyrinth is much smaller than normal. The abnormality of many mutant embryos can be reversed by making chimeras with wild-type tetraploid cells, implying that functions of the genes are required within the trophoblast compartment but not the vascular endothelium [3]. *Unc5b* seems to be the only gene that has been reported not to affect trophoblast development and vasculature in the yolk sac and embryo, but instead regulates angiogenesis in the labyrinth [12].

*Mst1* and *Mst2* (thereafter called *Mst1/2*), encoding two highly conserved Ser/Thr kinases, are the mammalian homologues of *Drosophila Hpo,* which controls organ size by negatively regulating cell proliferation and apoptosis [13]. Tissue-specific deletion of *Mst1/2* in mice results in tissue-specific tumorigenesis and confirms their conversed physiological roles as tumor suppressors [14], [15], [16]. Loss of function studies of *Mst1* mice also reveal novel functions of *Mst1* in T cell development and trafficking [17], [18], [19], [20]. Universal deletion of *Mst1/2* in mice results in embryo lethality at midgestation accompanied by impaired vascularization in labyrinth/yolk sac/embryo, neural tube unclosure and severe growth retardation [21]. However, the cellular and molecular mechanisms underlying the defects of *Mst1/2-*deficient embryonic and placental development remain to be elucidated.

In this study, we evaluated the effect of combined *Mst1/2* deficiency on mouse placental development during midgestation using conventional and endothelial-specific *Mst1* and *Mst2* knockout mouse models. We found that universal deletion of *Mst1*/*2* severely impaired trophoblast cell differentiation by down regulating *Mash2* expression and impeded labyrinth morphogenesis at midgestation. We also showed that endothelial-specific deletion of *Mst1/2* led to impaired placental labyrinth vasculature and embryonic lethality, but had neither obvious effects on vasculature in both yolk sacs and embryo proper nor endocardium development before E11.5. Our findings reveal a novel role for *Mst1/2* in trophoblast cell differentiation and labyrinthine vasculature at midgestation and suggest that *Mst1/2* control labyrinth morphogenesis in trophoblast- and fetal endothelial-dependent manners.

## Results

### High Expression of *Mst1*/*2* in Developing Mouse Placentas

**Figure 1:**
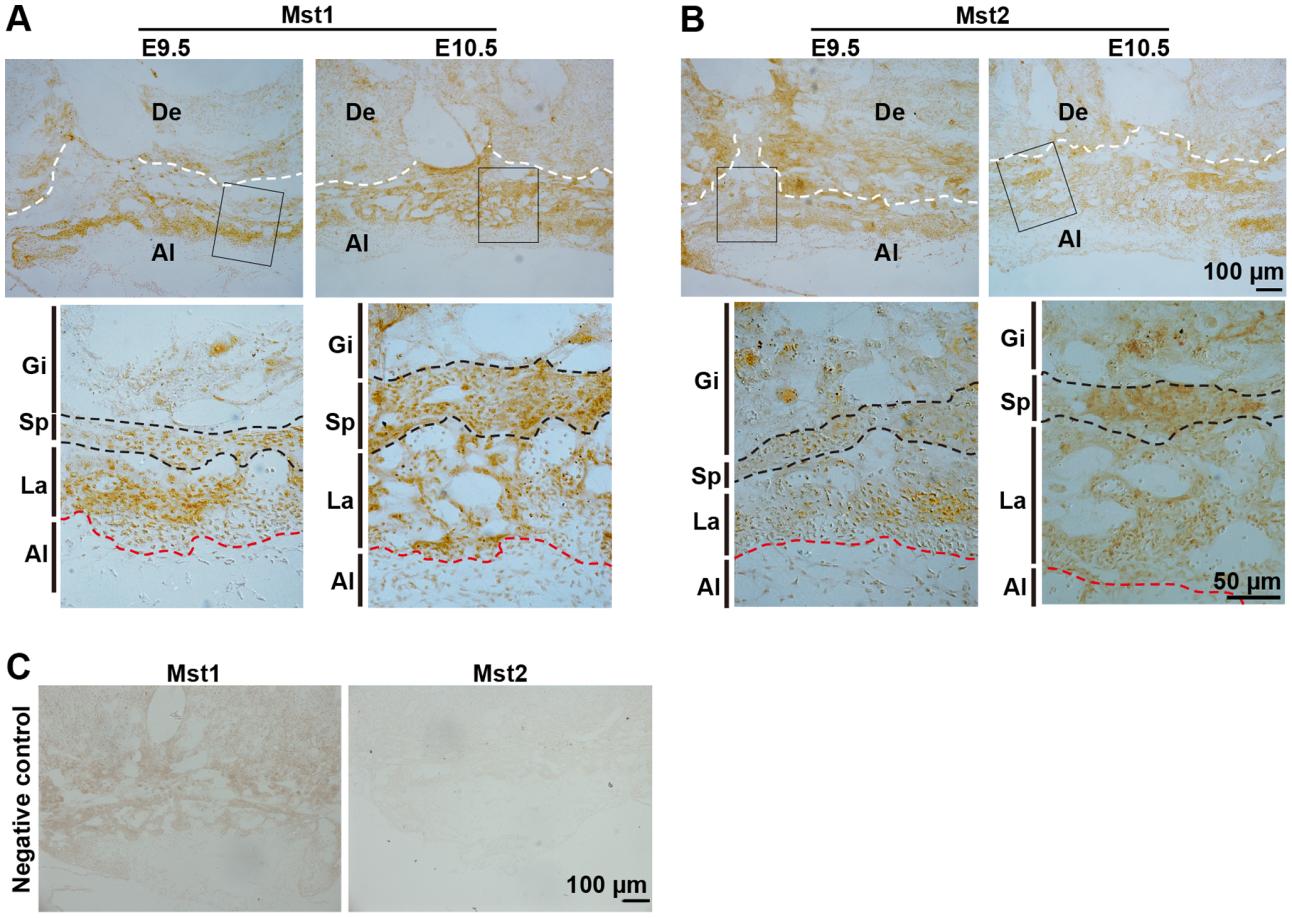
Expression of *Mst1* and *Mst2* in E9.5 and E10.5 mouse placentas. (A and B) Images of E9.5 and E10.5 placenta sections of wild type (129) mouse embryos immunohistochemically stained with anti-Mst1 (A) and anti-Mst2 (B) antibodies. High magnification images of the cropped regions in (A) and (B) are shown under each panel, respectively. White dashed lines indicate the boundary between the giant trophoblast cell layer and maternal decidua and red dashed lines designate the boundaries between the labyrinth and allantois. Black dashed lines mark the spongiotrophoblast layer. (C) The negative control for immunohistochemistry. Images of placenta sections from E10.5 *Mst1* ^−/−^ *Mst2* ^+/−^ (left) or *Mst1* ^+/−^ *Mst2* ^−/−^ (right) embryos stained with anti-Mst1 or anti-Mst2 antibody, respectively. Images are representative sections from at least three placentas from three pregnant mice per group. Al, allantois; De, decidua; Gi, trophoblast giant cells; La, labyrinth; Sp, spongiotrophoblast layer.

To study the function of *Mst1* and *Mst2* genes in mouse placental development, we first examined the spatiotemporal expression pattern of *Mst1/2* in developing mouse placenta at E9.5 and E10.5 by immunohistochemistry. We observed the abundant expression of *Mst1* in all three trophoblast layers (trophoblast giant cell, spongiotrophoblast and labyrinth layers) of E9.5 and E10.5 placentas and a relatively lower expression in the surrounding tissues such as the allantois (Fig. 1A). An expression pattern similar to that of *Mst1* was also observed for *Mst2* (Fig. 1B). Our data is consistent with the result of Northern blot analysis of *Mst1/2* expression in human tissues [22], [23]. The high and overlapped expression of *Mst1* and *Mst2* in mouse and human placenta suggests that they may play redundant but critical roles in placental development.

### Severely Impaired Trophoblast Differentiation and Labyrinth Morphogenesis in *Mst1/2* DKO Placentas

**Figure 2:**
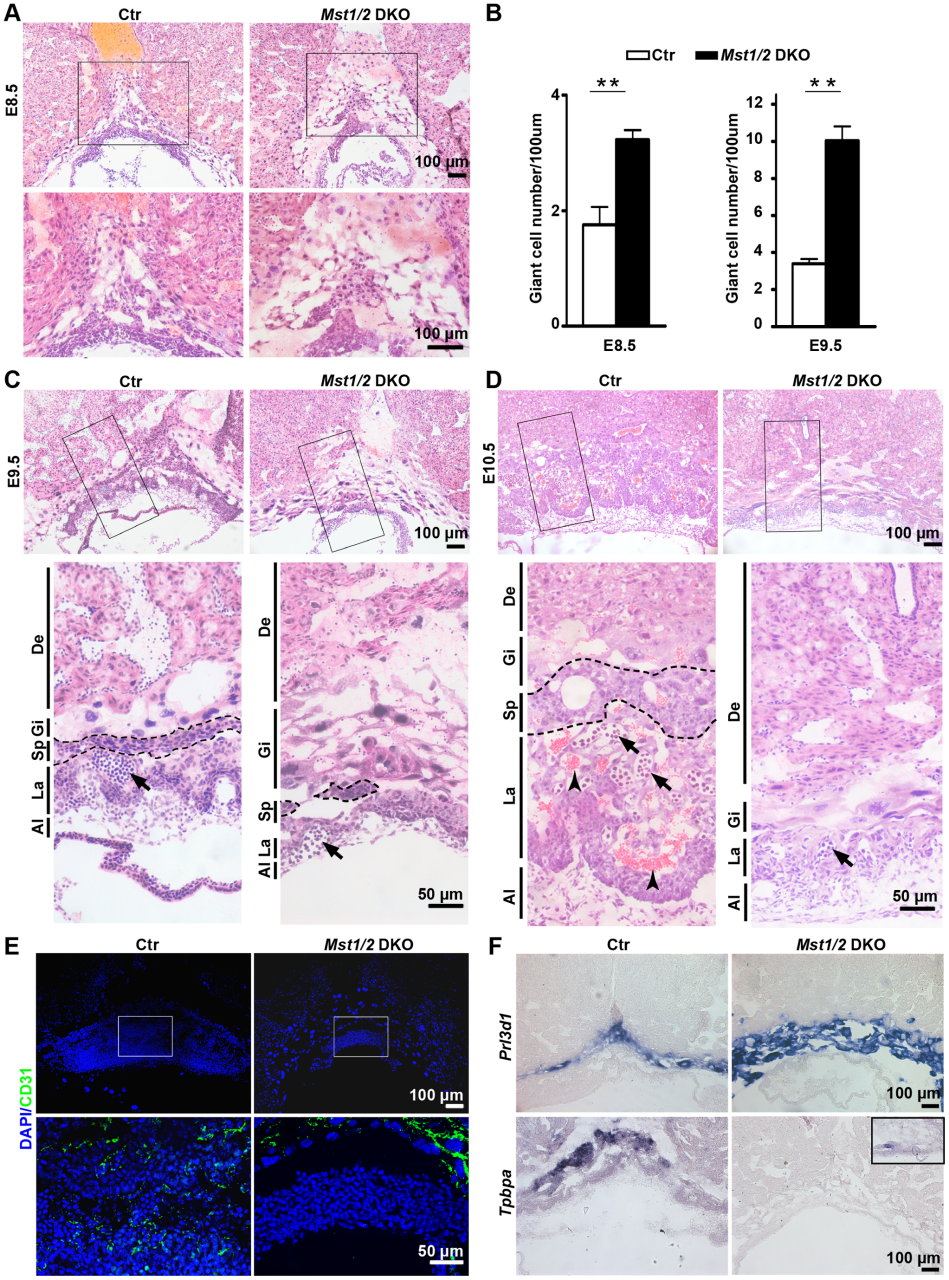
Impaired differentiation of trophoblast giant cells and spongiotrophoblast cells and labyrinthine development in the *Mst1/2 *DKO placenta. (A, C and D) Images of hematoxylin/eosin-stained placental sections of the control (Ctr) and *Mst1/2* DKO embryos at E8.5 (A), E9.5 (C) and E10.5 (D). (B) Statistical analysis of TGC in the ectoplacental cone of E8.5 and E9.5 placental sections. Data are expressed as the means ± SEM (three sections/placenta, three placentas from three pregnant mice per group). ** *P*<0.01. (E) CD31-immunofluoresence staining of cryosections of E9.5 Ctr and *Mst1/2* DKO placentas. High magnification images of the cropped region in (A), (C), (D) and (E) are shown under each panel, respectively. Dashed lines indicate spongiotrophoblast layers. Arrows and arrowheads indicate fetal nucleated and maternal enucleated red blood cells, respectively. (F) RNA *in situ* hybridization of E9.5 placental cryosections with a probe for the *Prl3d1* gene which specifically marks TGC (upper panel) and a probe for the *Tpbpa* gene which marks spongiotrophoblast cells (lower panel). Inset in lower panel is an enlarged photo to show the existence of a weaker *Tpbpa* signal in *Mst1/2* DKO placentas. Images are representative sections from at least three placentas from three pregnant mice per group. Al, allantois; Ctr, control; De, decidua; Gi, trophoblast giant cells; La, labyrinth; Sp, spongiotrophoblast layer.

To investigate the roles of *Mst1/2* in placental development, histopathological analyses of *Mst1* ^−/−^ *;Mst2* ^−/−^ (*Mst1/2 *DKO) placentas at E8.5, E9.5 and E10.5 were performed. The results revealed that normal trophoblast differentiation was severely impaired in *Mst1/2* DKO placentas from earlier stages. By E8.5 there were more trophoblast giant cells in the ectoplacental cone (EPC) of *Mst1/2* DKO placentas compared with the control (Fig. 2A and B). In E9.5 control placentas, the EPC was still obvious, and the trophoblast giant cells were presented as a discontinued single-cell layer while the spongiotrophoblast cells formed a multi-cell layer above the developing labyrinth layer. Fetal blood vessels invaded the chorionic plate and started branching into the developing labyrinth layer (Fig. 2C left panel). However, in E9.5 *Mst1/2 *DKO placentas, the EPC almost completely disappeared and the number of trophoblast giant cells dramatically increased (Fig. 2B and 2C right panel), forming a multi-cell layer while the prospective spongiotrophoblast layer was reduced to small pockets of cells (Fig. 2C right panel). The chorionic plate remained more compact than the control and lacked signs of fetal blood vessel invasion (Fig. 2C right panel), which was confirmed by immunofluorescent staining for CD31 (Fig. 2E), an endothelial cell marker. These results demonstrate that labyrinth morphogenesis in E9.5 *Mst1/2 *DKO placentas was also severely impaired. At E10.5, the labyrinth of control placentas continued to expand and formed a thick layer of labyrinth with a large number of fetal blood vessels filled with nucleated erythrocytes and maternal sinusoids containing enucleated erythrocytes (Fig. 2D left panel). In contrast, in *Mst1/2 *DKO placentas, no characteristic labyrinth layer was formed although fetal blood vessel invasion seemed to occur. Furthermore, the spongiotrophoblast layer almost disappeared in *Mst1/2 *DKO placenta at E10.5 (Fig. 2D right panel). To further confirm this phenotype, RNA in situ hybridization was performed. We observed a dramatic increase in the signal of *Prl3d1* (previously known as *PL1*, placental lactogen-1), which is a specific marker for trophoblast giant cells [30]. In contrast, the expression of *Tpbpa* (also known as 4311), a marker for spongiotrophoblast cells in the placenta and their precursors in the EPC [24], was nearly absent in *Mst1/2 *DKO placenta at E9.5 (Fig. 2F). Our studies demonstrate that *Mst1*/*2* are crucial for placental trophoblast differentiation and are key developmental regulators of mouse placental labyrinth morphogenesis.

### *Mst1*/*2* Deficiency does not Affect the Proliferation/Apoptosis of Trophoblast Giant Cells and Spongiotrophoblast Cells

**Figure 3:**
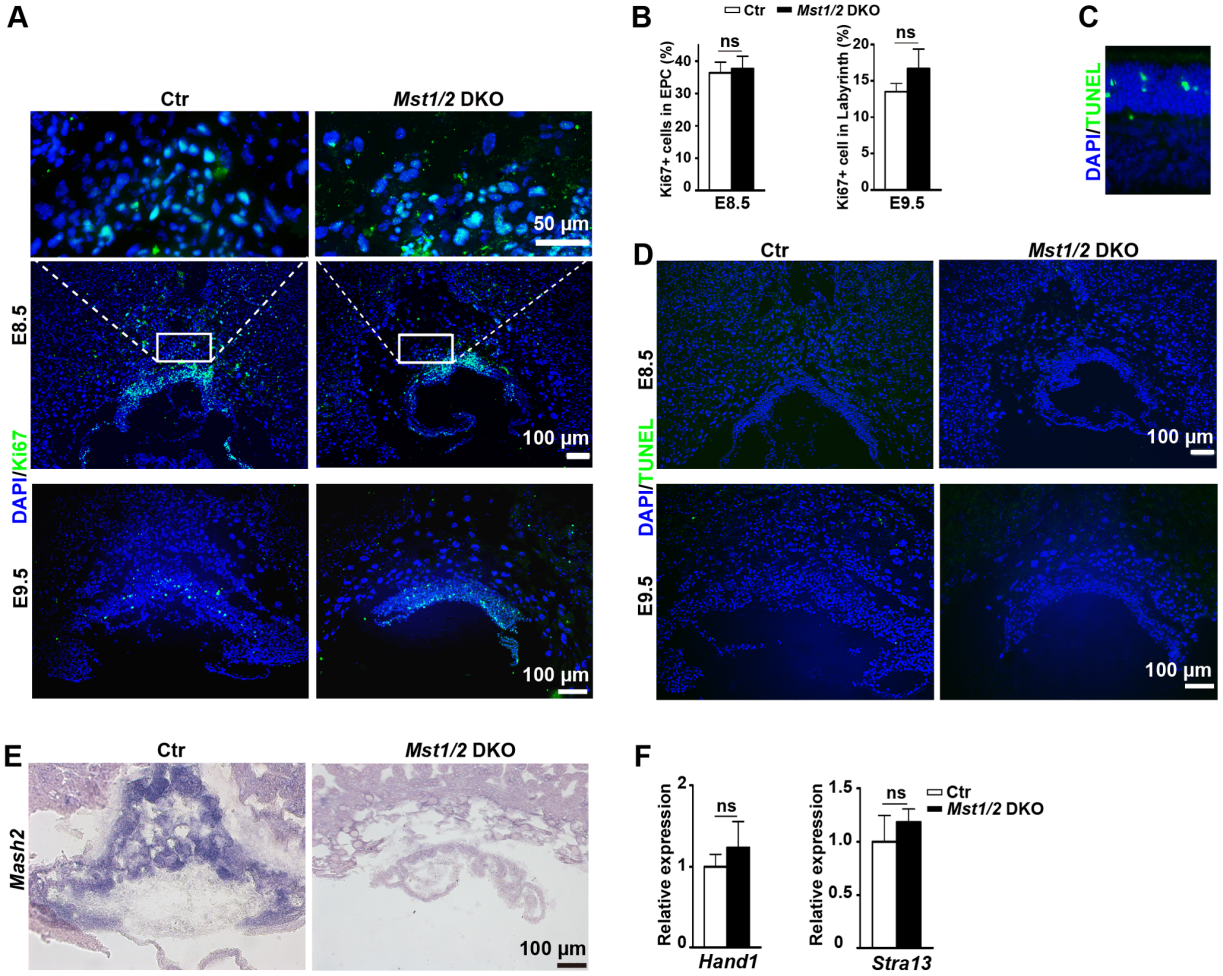
Downregulation of *Mash2* expression in *Mst1/2* DKO placentas. (A and D) Images of Ki67 immunofluoresence staining (A) and TUNEL assay (D) of the placental cryosections of the indicated genotypes and ages. High magnification images of the cropped region of the ectoplacental cone in (A) were shown above each panel respectively. (B) Statistical analysis of cell proliferation in the ectoplacental cone (left panel) and the labyrinth (right) of E8.5 or E9.5 placentas of the indicated genotypes. (C) Positive control of TUNEL assay with the *Dlic1* ^−/−^ retina section. (E) RNA in situ hybridization analysis of E9.5 placental cryosections with a probe of *Mash2* gene. (F) Quantitative RT-PCR analysis of *Hand1* (left panel) and *Stra13* (right panel) mRNA levels in E9.5 placentas of the indicated genotypes. (n = 3 for each group). Images in (A), (D) and (E) are representative sections from at least three placentas from three pregnant mice per group. Data in (B) and (F) are expressed as the means ± SEM, ns: not significant.

*Mst1/2* are well known for their roles in suppressing cell proliferation and promoting apoptosis [13]. Given the high expression of *Mst1/2* in mouse placentas, it is possible that *Mst1/2* regulate placenta development by controlling trophoblast cell proliferation and apoptosis. To test this possibility, we first evaluated cell proliferation of trophoblast giant cells and spongiotrophoblast cells in *Mst1/2* DKO placentas by Ki67 immunofluorescent staining. Results showed that there was almost no Ki67 signal in the trophoblast giant cell layer *of* both *Mst1/2 *DKO and control placentas at E9.5. The proliferation signals were mainly located in the labyrinth layer of *Mst1/2* DKO and control placentas and there was no significant difference between them (Fig. 3A bottom panel and B). Since trophoblast giant cells are mainly differentiated from the ectoplacental cone cells directly after implantation, we further evaluated cell proliferation in E8.5 *Mst1/2* DKO placentas. The analysis revealed that the proliferation signals in the EPC *of Mst1/2* DKO placentas were comparable to those of controls (Fig. 3A top and middle panels and B). These results suggest that the dramatic increase of trophoblast giant cells in *Mst1/2 *DKO placentas was not due to enhanced proliferation. Next, we further examined the apoptosis of placental trophoblast cells by TUNEL assay. No obvious apoptotic cells were found in both *Mst1/2 *DKO and control placentas at E9.5 and E8.5 (Fig. 3D). These observations indicate that the diminished number of spongiotrophoblast cells and increased number of trophoblast giant cells in *Mst1/2* DKO placentas were not due to abnormal proliferation and apoptosis of trophoblast cells.

### *Mst1*/*2* are Required for Maintaining *Mash2* Expression in Placentas

Both the secondary trophoblast giant cells and spongiotrophoblast cells differentiate from precursors in the EPC [2], [4]. The differentiation towards TGC lineage is thought of as a ‘default’ differentiation pathway in the absence of trophoblast stem cell maintenance. The differentiation to spongiotrophoblast cells requires the activation of certain signal pathways to suppress the ‘default’ differentiation of trophoblast giant cells [4]. The basic helix-loop-helix transcriptional factor, Mash2, is expressed in the ectoplacental cone cells and is required for suppressing the formation of trophoblast giant cells. Targeted deletion of *Mash2* resulted in a reduction of mouse ectoplacental cone cells, loss of the subsequent spongiotrophoblast layer, and a concurrent expansion of trophoblast giant cells [5], [6]. The EPC almost completely disappeared in *Mst1/2* DKO placentas (Fig. 2C right panel). To understand the molecular mechanism(s) by which *Mst1/2* regulate trophoblast differentiation, we examined the expression of *Mash2* in *Mst1/2* DKO placentas by RNA in situ hybridization. The results showed the *Mash2* expression was dramatically reduced in *Mst1/2* DKO placentas (Fig. 3E).

The bHLH transcription factors *Hand1* and *Stra13* can promote TGC differentiation [7]. Therefore, we also evaluated the effect of *Mst1/2* deficiency on the expression of *Hand1 and Stra13* genes by quantitative RT-PCR. We found that mRNA levels of *Hand1 and Stra13* in *Mst1/2* DKO placentas were comparable to those of controls (Fig. 3F). Taken together, our results suggest that impaired *Mash2* expression could be the reason behind the abnormal differentiation of trophoblast cells in *Mst1/2* DKO placentas.

### Endothelial-specific Deletion of *Mst1* and *Mst2* Impairs Placental Labyrinth Vascularization and Leads to Embryonic Lethality

**Figure 4:**
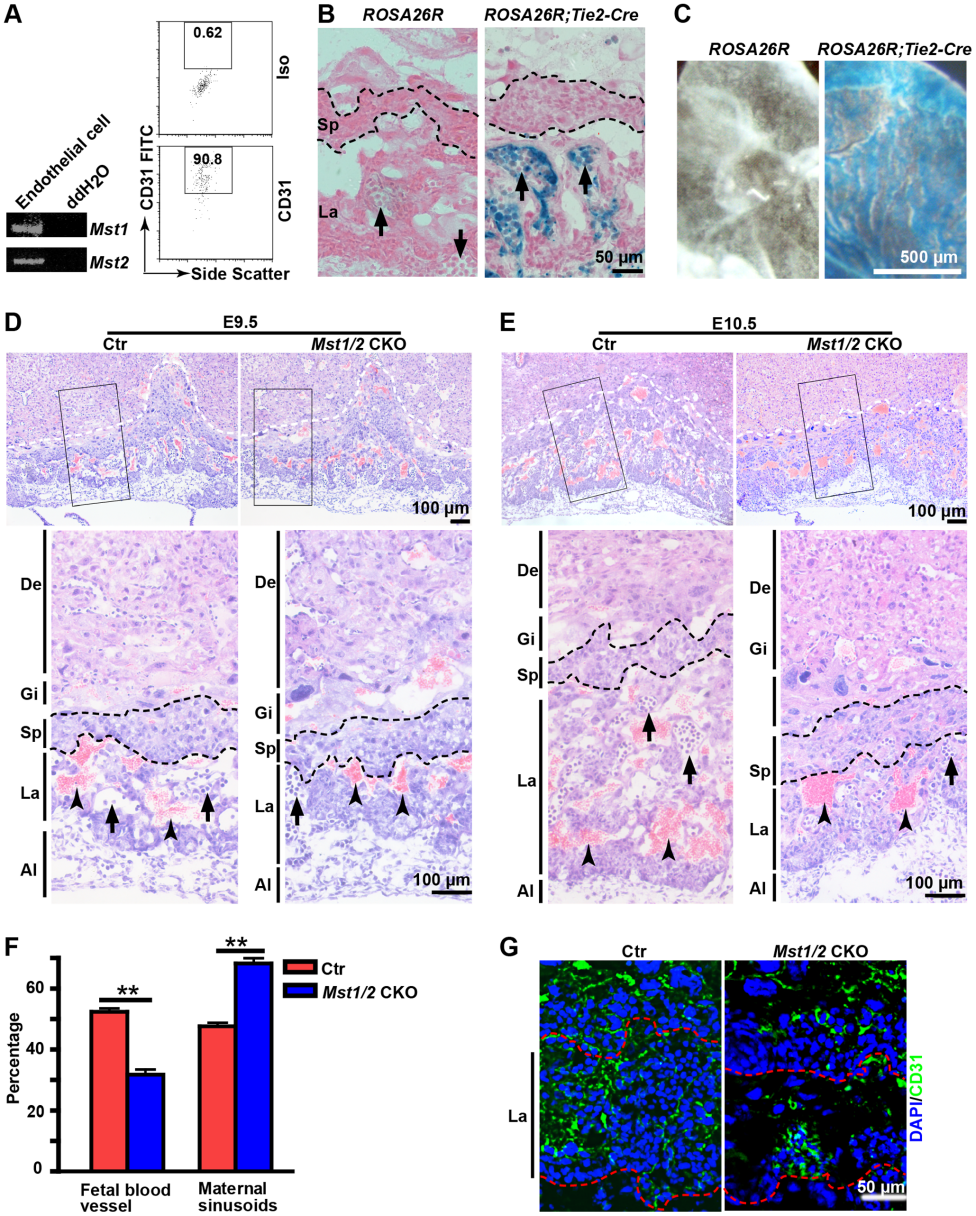
Defective labyrinthine vascularization/morphogenesis in *Mst1/2* CKO placentas. (A) RT-PCR analysis of *Mst1/2* expression in the purified endothelial cells from E10.5 wild type embryos and yolk sacs (left panel) and Flow cytometry analysis of CD31 expression of the purified endothelial cells (right panel). (B) Images of X-gal-stained E9.5 placental cryosections with indicated genotypes. Note: *β-gal* gene was expressed on blood vessels containing nucleated fetal red blood cells. Black dashed lines indicate the spongiotrophoblast layer. (C) Images of whole-mount X-gal-stained E9.5 yolk sacs with indicated genotypes. (D and E) Images of HE-stained cryosections of Ctr and *Mst1/2 *CKO placentas at E9.5 (D) and E10.5 (E). High magnification images of the cropped region in (D) and (E) are shown under each panel, respectively. The white dashed lines are the boundaries between maternal decidua and trophoblast giant cell layer. Black dashed lines mark the spongiotrophoblast layer. (F) Statistical analysis of the percentage of fetal blood vessels and maternal sinusoids in the labyrinth layer of control and *Mst1/2* CKO placentas at E10.5. Data are expressed as the means ± SEM (three sections/placenta, three placentas from three pregnant mice per group, ** *P*<0.01). (G) CD31 immunofluoresence staining of cryosections of E10.5 control and *Mst1/2 *CKO placentas. The labyrinth layer is defined by the red dashed lines. Arrows and arrowheads indicate fetal nucleated and maternal enucleated red blood cells, respectively. Images are representative sections from at least three placentas per group. Al, allantois; Ctr, control; De, decidua; Gi, trophoblast giant cells; Iso, Isotype control; La, labyrinth; Sp, spongiotrophoblast layer.

The abnormal phenotype of smaller labyrinth in many mutant embryos can be reversed by the provision of wild-type trophoblast in tetraploid chimeras [3]. Although this strongly suggests that placental trophoblast cells play important roles in labyrinthine morphogenesis and vascularization, the endothelial cells derived from the allantois are indispensable for the same processes [2], [3]. *Mst1/2* DKO embryos displayed failed fetal blood vessel invasion and branching in placental labyrinth (Fig. 2C and D), impaired yolk sac vascular development and severe cardiovascular defects (data not shown and Oh, et al [21]). These results could indicate that endothelial function of *Mst1/2* is required for labyrinthine vascularization as well as vasculature of yolk sac and cardiovascular development. These also could be the secondary effects of *Mst1/2* DKO placenta developmental defects resulting from impaired trophoblast differentiation. To distinguish between these two possibilities, we first verified the expression of *Mst1/2* in endothelial cells (Fig. 4A). To ascertain that endothelial function of *Mst1/2* was required for labyrinthine vascularization and placenta morphogenesis, we specifically deleted the *Mst1* and *Mst2* genes in endothelial cells using an *Tie2-Cre* transgenic mouse line [25], in which endothelial-specific Cre-expression was previously demonstrated in E9.5 embryos [26]. We have further confirmed Cre-expression in placentas and yolk sacs at E9.5 (Fig. 4B and C) and E10.5 (data not shown) with the ROSA26 Cre reporter mice. *Mst1^fl/fl^;Mst2^−/−^;Tie2*-Cre (thereafter called *Mst1/2 *CKO) mice were generated by crossing *Mst1* ^fl/fl^;*Mst2* ^−/−^ mice with *Mst1* ^+/fl^;*Mst2* ^−/−^;*Tie2-Cre* mice. Our studies showed that *Mst1* ^fl/fl^;*Mst2* ^−/−^, *Mst1* ^+/fl^;*Mst2* ^−/−^ and *Mst1* ^+/fl^;*Mst2* ^−/−^;*Tie2-Cre* mice derived from the cross did not display overt abnormality, therefore they were used as the control. However, in the total 130 progeny genotyped at postnatal day 7, no *Mst1/2 *CKO mice were identified, suggesting embryonic or neonatal lethality of *Mst1/2 *CKO mice.

In placental labyrinth of *Tie2-Cre* transgenic mice, the Cre activity could be easily detected at E9.5 in endothelial cells derived from the *Tie2-Cre* transgenic embryo (Fig. 4B). We hypothesized that endothelial-specific deletion of *Mst1/2* affected labyrinthine vascularization and placenta morphogenesis. To test our hypothesis, we performed a histopathological analysis of *Mst1/2 *CKO placentas. Consistent with the *Tie2-Cre* expression pattern in placentas, no obvious defects of the trophoblast giant cell layer and prospective spongiotrophoblast layer were observed in E9.5 *Mst1/2 *CKO placenta. The invasion and branching of fetal blood vessels in the labyrinth of *Mst1/2 *CKO placenta at E9.5 were similar to the control except that the mutant labyrinth seemed a little thinner (Fig. 4D). At E10.5, the labyrinth of control placentas expanded dramatically and the branch morphogenesis continued to form complicated vascular structures containing both maternal sinusoids and fetal blood vessels. However, the labyrinth of *Mst1/2 *CKO placentas was much thinner and contained fewer fetal blood vessels than the control placenta (30% of total blood vessels in the mutant versus 55% in the control (Fig. 4E and F). Lack of penetration of the fetal blood vessels in the labyrinth of *Mst1/2 *CKO placentas was also confirmed by immunofluorescent staining with anti-CD31 antibody (Fig. 4G). All these results suggest that the functions of *Mst1*/*2* in endothelial cells are required for labyrinthine vascularization and morphogenesis.

**Figure 5:**
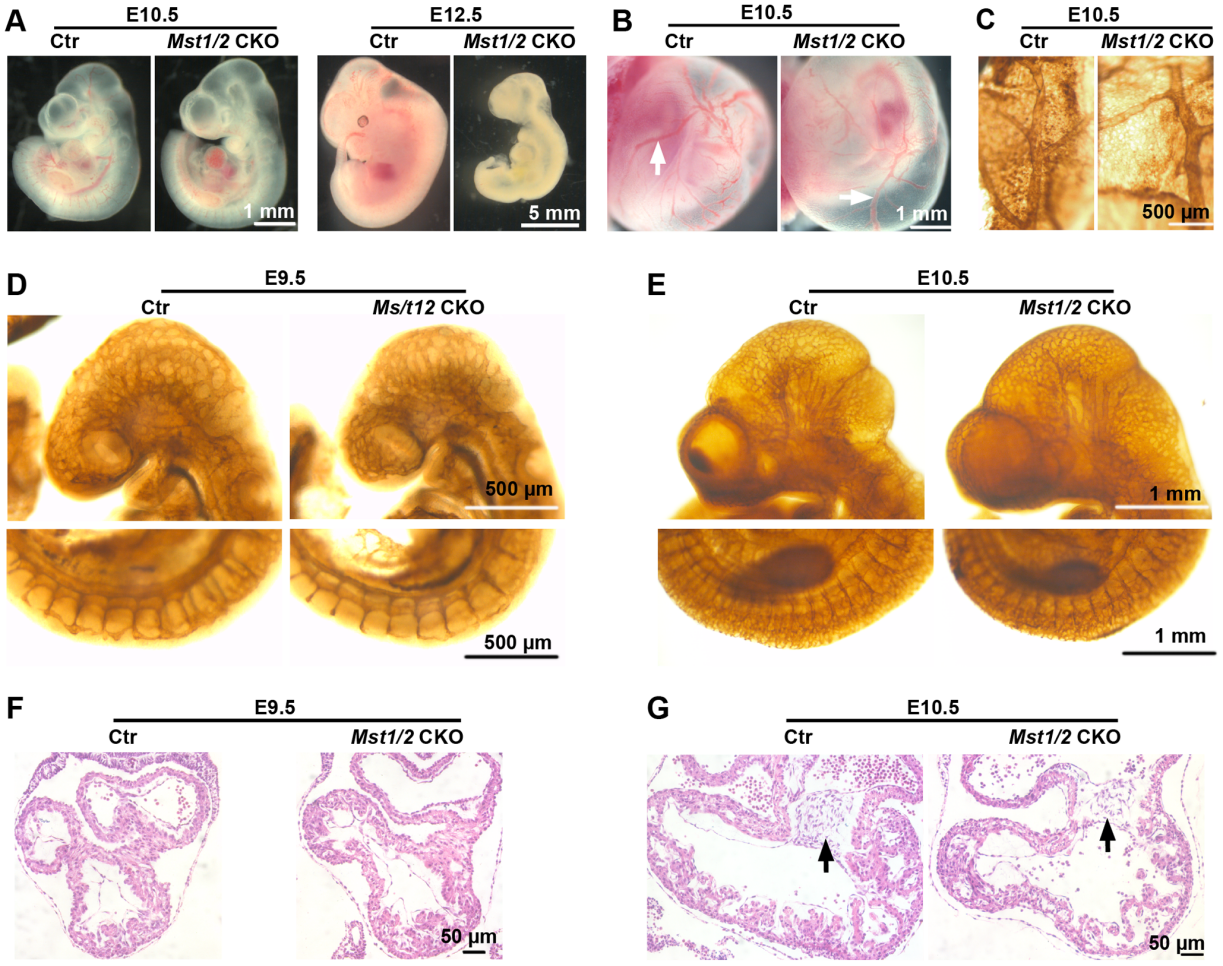
The effects of endothelial-specific deletion of *Mst1/2* CKO on cardiac and vascular development during midgestation. (A and B) Photographs of freshly dissected embryos proper (A) and yolk sacs containing embryos (B) with indicated genotypes and ages. Large vitelline blood vessels (white arrows) are observed on both the control and mutant yolk sacs. (C-E) Vasculature in E10.5 yolk sacs (C), head and intersomitic regions of E9.5 (D) and E10.5 (E) embryos of the indicated genotypes visualized by whole-mount anti-CD31 immunohistochemistry. (F and G) HE-stained transverse paraffin sections of E9.5 (F) and E10.5 (G) hearts of the indicated genotypes. Black arrows indicate atrioventricular cusions. Images are representatives from at least three embryos per group.

Consistent with the defect of labyrinthine vasculature and placenta morphogenesis, *Mst1/2 *CKO mice died during embryonic development. Detailed analysis revealed that no *Mst1/2 *CKO mice were viable at E12.5 while all the *Mst1/2 *CKO embryos were viable at E11.5 (Fig. 5A and Table 1). These data suggest that the endothelial cell-autonomous function of *Mst1/2* is required for embryonic survival.

**Table 1:** Numbers of *Mst1/2* CKO and Ctr offsprings.

Age	Ctr	Mst1/2 CKO	ND	Total
E9.5	17	7	0	24
E10.5	16	6	0	22
E11.5	12	4	0	16
E12.4–14.5	16	6*	2	24

Cross: *Mst1* ^+/fl^;*Mst2* ^−/−^ *;Tie2-Cre* X *Mst1* ^fl/fl^;*Mst2* ^−/−^.

Ctr includes: *Mst1^+/fl^;Mst2^−/−^;Tie2-Cre*, *Mst1^fl/fl^;Mst2^−/−^*, *Mst1^+/fl^;Mst2^−/−^*.

ND: not determined.

*dead embryos.

### Endothelial-specific Deletion of *Mst1*/*2* does not Affect Vasculature and Endocardium Development in the Midgestation Mouse Embryos

Endothelial cells are the first components of the cardiovascular system to undergo differentiation and are required for the whole process of vascular patterning [27]. We had previously confirmed *Tie2-Cre* activation in E9.5 mouse embryos and reported multiple cardiac and vascular defects of *Adam 10^fl/fl^; Tie2-Cre* mouse embryos at E9.5 [26]. Since *Mst1/2* DKO mice displayed impaired yolk sac vascular development and severe cardiovascular defects (data not shown and Oh, et al [21]), we assumed that endothelial-specific deletion of *Mst1*/*2* would result in defects of the yolk sac vasculature and the embryonic cardiovascular system, which might lead to the embryonic lethality of *Mst1/2 *CKO mice. However, we found that the yolk sac vasculature of *Mst1/2 *CKO embryos at E10.5 appeared normal and large vitelline blood vessels were as well developed as the control (Fig. 5B). This observation was further confirmed by whole mount immunohistochemical staining with anti-CD31 antibodies. The results showed that at E10.5 major blood vessels and a well-branched capillary network, which were similar to the control, were present in *Mst1/2* CKO yolk sac (Fig. 5C). Furthermore, well-developed and defined capillary networks were also apparent in the head and intersomitic regions of both *Mst1/2 *CKO and control embryos proper at E9.5 and E10.5 (Fig. 5D and E). These results demonstrate that endothelial-specific deletion of *Mst1*/*2* does not affect yolk sac and embryonic vasculature in mice.

Endothelial cells are also critical for cardiac development [28]. In order to determine the effect of endothelial-specific deletion of *Mst1*/*2* on cardiac development, we performed histopathological analysis of serially sectioned *Mst1/2 *CKO embryonic hearts. Our analysis showed that at E9.5 the size and structure of the *Mst1/2 *CKO heart was very similar to that of the control (Fig. 5F). The endocardium was well developed and the atrioventricular (AV) cushion started to develop. However, at E10.5 the *Mst1/2* CKO heart had a smaller atrioventricular (AV) cushion which contained less cells and ventricular trabeculation was underdeveloped (Fig. 5G). These results suggest that endothelial cell intrinsic function of *Mst1*/*2* was not required for endocardium development of embryo heart before E9.5, but endothelial-specific *Mst1*/*2* deficiency might either affect mouse heart development after E9.5 or just be a secondary effect of the placental defect of the *Mst1/2* CKO embryo (see discussion).

## Discussion

In the studies reported here we have demonstrated that universal deletion of *Mst1/2* impairs placental development by interfering with trophoblast differentiation and blocking labyrinthine vascularization. We found a dramatic increase in TGC number and almost a complete lack of spongiotrophoblast cells in *Mst1/2* DKO placentas, which phenocopies the mouse *Mash2* mutant [5], [6]. Furthermore, we have shown that the expression of *Mash2,* which inhibits the default TGC differentiation, is down regulated at transcription level in *Mst1/2* DKO placentas while the expression of *Hand1* and *Stral3,* which promote TGC differentiation, are not significantly affected at mRNA levels. These results suggest that *Mst1/2* may regulate trophoblast differentiation and placental morphogenesis by activating the *Mash2* expression. YAP/Yki are well-defined downstream targets of *Hpo/Mst* pathway. It is well known that inactivation of the *Hpo/Mst* pathway leads to nuclear entrance and activation of YAP/Yki transcription co-activators in phosphorylation-dependent and phosphorylation-independent mechanisms [29]. Because the *Mash2* expression was down regulated in *Mst1/2*-deficient trophoblast cells, we infer that YAP cannot act as a transcription co-activator here. Given the above results and analysis, we propose that YAP may function as a transcription co-repressor, rather than a transcription co-activator, in *Mst1/2*-mediated activation of *Mash2* expression in trophoblast cells. Consistent with our proposal, YAP was reported to be a co-transcriptional repressor for the osteocalcin gene expression *in vivo* [30]. However, it is also possible that *Mst1/2* regulates *Mash2* expression by modulating the activity of an unidentified and/or trophoblast-specific transcriptional repressor or activator. In line with this possibility, we have recently found that *Mst1* regulates Foxp3 expression through phsphorylating and stabilizing transcriptional factors, Foxo1/3 [31]. It is worthy to perform further experiments using trophoblast cell lines to verify these possibilities.

We have also shown that endothelial-specific function of *Mst1/2* is required for optimal vascular development of labyrinth although it is dispensable for the vasculature of yolk sac/embryo proper and early endocardium development. The placenta is one of the more complex vascularized tissues in mammals. The fetal-placental vascular system circulates fetal blood and interdigitates with trophoblast sinuses filled with maternal blood. The signaling between the extra-embryonic tissue and the fetal-placental vessels is required for vascularization within the placental labyrinth [3], [32]. Almost all previous mutational analyses of dozens of genes required for this communication demonstrate that patterning by the trophoblast cells is an essential guide for proper growth of the fetal vessels into and within the labyrinth layer and conclude that all these genes exert their effects through the extraembryonic trophectoderm [1], [4]. The exception is the study of *Unc5b* gene whose expression is restricted to endothelial cells in the placenta. *Unc5b* deficiency results in a dramatic reduction of arteriole numbers in the placenta but does not affect the vasculature in yolk sac and embryo proper, emphasizing that vascular signaling pathways on the fetal-placental side of the equation should not be ignored [12], [33]. Here we show that universal deletion of *Mst1/2* impaired proper trophoblast differentiation and almost completely blocked labyrinthine vascularization while endothelial-specific *Mst1/2* deficiency only resulted in a much weaker vascular defect of the labyrinth. The differences of labyrinthine developmental defects between *Mst1/2* DKO and *Mst1/2 *CKO placentas indicate a critical role of *Mst1/2* in labyrinthine trophoblast development. These results also suggest that *Mst1/2* genes may control placental labyrinth vasculature through regulating signals from both extraembryonic trophectoderm and fetal-derived endothelial cells, in other word, in trophoblast- and fetal endothelial-dependent manners.

Based on the fact that endothelial specific function of *Mst1/2* is required for optimal vascular development of labyrinth but dispensable for the vasculature of yolk sac/embryo proper and early endocardium development, we suggest that impaired vasculature in yolk sac/embryo proper and endocardium development in *Mst1/2 *DKO embryos reported by Oh, et al [21] may be the secondary consequence of very severe developmental defects of the *Mst1/2 *DKO placenta. Our unpublished study also reveals that neural-specific deletion of *Mst1/2* does not affect embryonic neural development and viability of *Mst1* ^fl/fl^;*Mst2* ^−/−^;*Nestin-Cre* mice (Du and Tao, unpublished data), demonstrating that failed closure of cranial neural tube of *Mst1/2 *DKO embryos showed by Oh, et al [21] may be also a secondary effect of growth retardation of the embryos. At E10.5 the *Mst1/2 *CKO hearts displayed a smaller AV cushion containing less cells and impaired ventricular trabeculation (Fig. 5G). Impaired AV cushion development could be an intrinsic defect of endothelial-specific *Mst1/2* deficiency in endothelial-mesenchymal cell transition. However, we cannot rule out the possibility that this phenotype is a secondary effect of placental defect of the mutant embryo. Impaired ventricular trabeculation may not be related to endothelial function of *Mst1/2*. It has been shown previously that deletion of *Yap,* which is negatively regulated by *Mst1/2* signaling, in the embryonic mouse heart impedes cardiomyocyte proliferation, causing myocardial hypoplasia and lethality at E10.5 [34] while inactivation of *Hpo* pathway by specifically deleting *Salv* leads to overgrowth of the heart with elevated cardiomyocyte proliferation [35]. Therefore, we reason that impaired ventricular trabeculation of *Mst1/2 *CKO hearts is probably a secondary consequence of the placental defect of the mutant embryo.

The results from our studies of *Mst1/2 *DKO and *Mst1/2 *CKO mice also suggest that placenta defects may be the primary reason leading to embryonic lethality of *Mst1/2 *DKO mice. There are two commonly used approaches to verify the contribution of placenta abnormalities to the embryonic lethality phenotype of mutant mice. One is tetraploid aggregation assay by which wild type tetraploid cells contribute exclusively to the trophoblast cells of the placenta and extra-embryonic endoderm, whereas mutant diploid cells can contribute to all the structures of the fetus and to the extraembryonic mesoderm including the mesoderm of the yolk sac, the allantoic mesoderm and the fetal blood vessels of the placenta [1]. The other approach is to apply specific Cre lines, such as the *Sox2* or *Mox2* transgenic Cre lines, to inactivate a gene only within epiblast cells that give rise to the entire embryo proper as well as to the extraembryonic mesoderm that forms the fetal vasculature in the placental labyrinth but not in the trophoblast of the placenta [36], [37]. However, both methods will result in a labyrinth with mutant fetal endothelial cells derived from the allantoic mesoderm. These two methods are not suitable for verifying the contribution of placenta abnormalities to the embryonic lethality phenotype of *Mst1/2 *DKO mice because we have shown that endothelial-specific deletion of *Mst1/2* caused placenta defects and embryonic lethality. Further studies using trophoblast-specific *Mst1/2* knockout mice are needed to solve this problem.

## Materials and Methods

### Mice

The generation of *Mst1* ^−/−^, *Mst1* ^fl/fl^ and *Mst2* ^−/−^ mice was described previously [17], [31]. *Mst1/2* DKO mice were maintained in 129 genetic background. The *Tie2-Cre* transgenic mouse line was kindly provided by Dr. X. Yang [25] and *Mst1/2* CKO mice were maintained in C57Bl6/129 mixed genetic background. The *Rosa26R* reporter mouse line was obtained from Jackson laboratory (strain: B6.129S4-Gtrosa26 tm1Sor; stock # 003474). The progeny were genotyped respectively as previously described [25], [31]. This study was carried out following the general guidelines published by the Association for Assessment and Accreditation of Laboratory Animal Care. The Animal Care and Use Committee of the Institute of Developmental Biology and Molecular Medicine at Fudan University approved all protocols used in animal experiments (Permit Number: 2007010).

### Histological, Immuonhistochemical and Immunofluorescent Analyses

Histological analysis was performed using the standard procedure.

For cryosections, dissected tissues or embryos were fixed for 30 minutes in 4% PFA followed by dehydration in 30% sucrose solution for two hours, and then embedded in OCT (Richard-Allan Scientific) and frozen in liquid nitrogen-cooled isopentane. Frozen sections were collected at 7 µm.

For immuonhistochemical and Immunofluorescent analyses, cryosections were stained with antibodies against the following proteins respectively: Mst1, Mst2 (Abcam 51134 and 52641), Ki67 (Novocastra Laboratories NCL-Ki67p) and CD31 (BD 558736) following the standard protocols [38].

Whole mount staining with anti-CD31 antibody (BD 550274) was performed as described by Takahashi, et al. [39] except for that signals were visualized in a DAB-color developing solution without NiCl_2._ Horseradish peroxidase (HRP)-conjugated mouse anti-rat IgG second antibodies were from Santa Cruz.

### TUNEL Assay

TUNEL assay was performed as described by Kong, et al [40]. Terminal Deoxynucleotidyl Transferase (NEB M0252V), dUTP-biotin (Roche 11093070910) and avidin-FITC (Invitrogen SA1001) were purchased from New England Biolabs, Roche Applied Science and Invitrogen, respectively.

### X-gal Staining

X-gal stainings for whole mount and cryosections were performed as previously described [41], [42] except for that samples for cryosections were first fixed and dehydrated in PBS solution containing 0.2% PFA and 30% sucrose overnight before being embedded in OCT and sectioned at 25 um.

### RNA in situ Hybridization

To generat RNA probes for RNA in situ hybridization**,** cDNA fragments for *Tpbpa, Prl3d1* (*PL1*) and *Ascl2* cDNA fragments were obtained by RT-PCR from the E9.5 *wild type* mouse placenta RNA using the following primers: *Tpbpa-*Forward: 5′-AGGATAAAGAAGTTCTCATA-3′; *Tpbpa-*Reverse: 5′-TGGCTGTGGTTTGTTTTC CTCCTC-3′; *Prl3d1*-Forward: 5′-GTTTGGCTGAACTGCTCCATAATAC-3′; *Prl3d1* -Reverse: 5′-AACTCGGCACCTCAAGACTTTG-3′; *Mash2-*Forward:5′-GTGCAAA CGTCCACTTCCCACC-3′; *Mash2-*Reverse:5′-TGCTTTCCTCCGACGAGTAGGC-3′.

The PCR products were then cloned into T-vectors and verified by DNA sequencing. Digoxigenin (DIG)-labeled riboprobes were prepared using the DIG RNA-labeling Kit (Roche 11175025910) according to the manufacturer’s instructions. RNA in situ hybridization was performed as previously described [43] with minor modifications. Briefly, placental cryosections were first incubated with indicated probes at 65°C for 36 hours, followed by washing and incubation with alkaline phosphatase-conjugated anti-DIG-antibody (Roche 11093274910). Signals were then visualized with BM purple (Roche 11442074001) as a substrate. Finally, the sections were counterstained with nuclear fast red (Shanghai Shenggong NB0671).

### Quantitative RT-PCR

RNA was extracted from placentas or purified endothelial cells with TRIzol (Invitrogen). Reverse transcriptase PCR and quantitative PCR were carried out as described previously [40]. The sequences of quantitative PCR primers are: Hand1-F, 5′-CATCGCCTACTTGATGGACGTG-3′; Hand1-R, 5′-CCCTTTAATCC TCTTCTCGCCG-3′; Unc5b-F, 5′-AGGACAGTTACCACAACCTACGCC-3′; Unc5b-R, 5′-ATGCCTCTCCAGGGTGAAAGTG-3′; Stra13-F, 5′-GGAAAAAGCAGT GGTTCTGGAGC-3′; Stra13-R, 5′-TCACGGGCACAAGTCTGGAAAC-3′; Gapdh-L1, 5′-TGTTCCTACCCCCAATGTGTCC-3′; Gapdh-R1, 5′-GGAGTTGCTGTTGAAGTC GCAG-3′.

### Isolation of Endothelial Cells and FACS Analysis

Endothelial cells were purified from E10.5 wild type embryos and yolk sacs as described before [44]. After purification, a fraction of cells were incubated with anti-CD31 antibody (Becton Dickinson, 558736) followed by FACS analysis for CD31 expression with a Calibur machine and FlowJo software (TreeStar).

### Micrographs and Statistical Analysis

Fluorescence micrographs were acquired using a Leica DMRXA2 fluorescence microscope equipped with a Leica DFC350FX camera. Histochemical micrographs were acquired using a Leica DMRXA2 fluorescence microscope equipped with a Leica DFC300FX camera. Images were processed using Adobe Photoshop.

Blood vessels in placenta labyrinth were counted as previously described [45]. Statistical analysis was conducted using unpaired t-test by GraphPad Prism 4. Results with p values less than 0.05 were considered significant.
